# Are Extensive Open Lung Resections for Elderly Patients with Lung Cancer Justified?

**DOI:** 10.3390/curroncol30060414

**Published:** 2023-06-05

**Authors:** Nikolaos Panagopoulos, Konstantinos Grapatsas, Vasileios Leivaditis, Michail Galanis, Dimitrios Dougenis

**Affiliations:** 1 Department of Thoracic Surgery, ‘Olympion’ General Clinic, 26443 Patras, Greece; npanag72@gmail.com; 2 Department of Thoracic Surgery, University Medicine Essen-Ruhrlandklinik, 45239 Essen, Germany; 3 Department of Cardiothoracic and Vascular Surgery, Westpfalz-Klinikum, 67655 Kaiserslautern, Germany; vleivaditis@westpfalz-klinikum.de; 4 Department of Thoracic Surgery, Inselspital, Bern University Hospital, University of Bern, 3012 Bern, Switzerland; michail.galanis@insel.ch; 5 Department of Cardiothoracic Surgery, Attikon University Hospital of Athens, 12462 Athens, Greece; ddougen@gmail.com

**Keywords:** lung cancer, non-small cell lung cancer, thoracotomy, anatomical lung resection, lobectomy, pneumonectomy, elderly

## Abstract

Background: Older patients with malignancies are more comorbid than younger ones and are usually undertreated only because of their age. The aim of this study is to investigate the safety of open anatomical lung resections for lung cancer in elderly patients. Methods: We retrospectively analyzed all patients who underwent lung resection for lung cancer in our institution and categorized them into two groups: the elderly group (≥70 years old) and the control (<70). Results: In total, 135 patients were included in the elderly group and 375 in the control. Elderly patients were more frequently diagnosed with squamous cell carcinoma (59.3% vs. 51.5%, *p* = 0.037), higher differentiated tumors (12.6% vs. 6.4%, *p* = 0.014), and at an earlier stage (stage I: 55.6% for elderly vs. 36.6%, *p* = 0.002). Elderly patients were more vulnerable to postoperative pneumonia (3.7% vs. 0.8%, *p* = 0.034), lung atelectasis (7.4% vs. 2.9%, *p* = 0.040), and pleural empyema (3.2% vs. 0%, *p* = 0.042), however, with no increased 30-day-mortality (5.2% for elderly vs. 2.7%, *p* = 0.168). Survival was comparable in both groups (43.4 vs. 45.3 months, *p* = 0.579). Conclusions: Elderly patients should not be excluded from open major lung resections as the survival benefit is not reduced in selected patients.

## 1. Introduction

From the early years of the last century, life expectancy has greatly increased [1]. Mortality due to lung cancer remains the leading cause of malignancy-related mortality worldwide, in both sexes [2]. Among lung cancer patients, non-small cell lung cancer (NSCLC) accounts for more than eighty percent of all cases and, like most other cancers, is considered a disease of advanced age. This increase in life span among Western countries, over the last decades, led to an increase in the number of elderly patients presenting NSCLC. NSCLC represents more than 80% of all lung cancer and in many cases, it is diagnosed in ages older than 70 years [3].

Older patients generally have poor cardiopulmonary reserve and are more comorbid than younger ones. For this reason, elderly patients with non-small cell lung cancer (NSCLC) are usually undertreated or treated conservatively only because of their age [4,5,6].

In recent years, improvements in video-assisted thoracic surgery (VATS) such as uniportal VATS and non-intubated VATS, as well as preoperative tumor localization (e.g., with the placement of wires preoperatively), have extended indications and possibilities for thoracic surgical resections, even for elderly patients. However, open lung resections are still performed whenever it is indicated [4,5,6,7].

The aim of this study was to investigate the safety of open anatomical lung resections in elderly patients in regard to younger ones and examine perioperative prognostic factors for survival among the elderly population.

## 2. Materials and Methods

### 2.1. Patient Selection and Perioperative Assessment

In this retrospective study, all patients with NSCLC who underwent anatomical lung resections in our institution from 2008 to 2013 were included. Demographic, clinical, and follow-up data were extracted from electronic medical records throughout a 5-year period. All patients were operated with open thoracotomy.

### 2.2. Preoperative Comorbidities

The following comorbidities were separately investigated considering their influence on survival: arterial hypertension, chronic obstructive pulmonary disease (COPD), diabetes mellitus, ischemic heart disease-coronary artery disease (IHD-CAD), acute myocardial infarction (AMI), atrial fibrillation, brain vascular disease, history of tuberculosis, history of previous malignancy, systemic autoimmune diseases, and preoperative chemo-/radiotherapy.

Patients with a history of cardiac comorbidities were in stable condition before the operation. For the preoperative clinical evaluation of the patients and the indication for surgery, the ERS/ESTS clinical guidelines were used [8,9].

Classification of COPD was made with GOLD criteria. In this category, patients with stage I-III COPD were included. No patient with COPD IV was identified in the study population [10].

All operations were carried out under general and epidural anesthesia with one-lung ventilation throughout lateral thoracotomy. All types of curative lung resections were performed, followed by radical systematic lymphadenectomy in all patients.

Daily administration of subcutaneous injection of low molecular weight heparin for thromboembolic prevention and at least three doses of perioperative prophylactic antibiotic therapy with ampicillin were given as standard protocol.

### 2.3. Postoperative Characteristics and Definition of Complications

Complications were observed up to the 30th postoperative day. All patients were monitored postoperatively and the complications presented were recorded. Postoperative complications comprised of cardiac (acute myocardial infarction, atrial fibrillation), respiratory (atelectasis, postoperative pneumonia, ARDS, respiratory failure requiring re-intubation) postoperative hemorrhage, reoperation (for hemorrhage or for other reason), acute renal failure, transient ischemic attack, prolonged air leak for more than seven days, empyema, bronchopleural fistula, and multiple organ failure (MOF). Postoperative pneumonia was diagnosed according to the European Perioperative Clinical Outcome (EPCO) criteria [11].

For the definition of major and minor complications, the Clavien-Dindo classification was used. Complications with a Clavien-Dindo grade ≥ III were classified as major and complications with Clavien-Dindo grade ≤ II were classified as minor [12].

### 2.4. Groups of Patients

A fixed age limit in the definition of “elderly” does not exist in the literature [4,13,14].

In our study, the patient population was divided into two groups: the elderly group and the control group. The elderly group included those patients aged 70 years or more. In the control group, patients with ages < 70 years were enrolled.

### 2.5. Inclusion and Exclusion Criteria

The study included all patients that were for NSCLC with an open method through thoracotomy.

Patients with the following criteria were excluded from the study:

- Patients aged < 18 years;
- Palliative surgical resections;
- Patients operated with thoracoscopy (VATS-video-assisted thoracic surgery) or other surgical access from thoracotomy;
- NSCLC resected by wedge resection;
- Patients with preoperative diagnosed pleural carcinomatosis or carcinomatous lymphangiosis.

Stage III lung resections were performed for patients with the clinical status: T1–2 N2, T3 N1, and T4 N0–1. Stadium IV was diagnosed postoperatively with the detection of tumor cells on the parietal pleura.

### 2.6. Data Collection and Statistical Analysis

The conduction of the study followed the revised Declaration of Helsinki. Before the beginning of the study, the study was approved by the local university ethics committee (Attikon University Hospital Institutional Scientific Ethical and Research Committee, No. 581/03-10-2022). Patients’ data were collected and stored in an anonymized database. Morbidity, mortality, and possible risk factors for postoperative morbidity were analyzed using cross tables, χ2 test, and Fisher’s exact test. Survival curves were estimated using the Kaplan–Meier method. For univariate survival comparison, the log-rank (Mantel–Cox) and Breslow (generalized Wilcoxon) tests were used. Univariate analysis was carried out using the Cox proportional hazards method. The selection of variables to enter the Cox regression model followed the backward elimination procedure, based on the likelihood of ratio statistic. Statistical significance was set for a *p* < 0.05. The statistical analyses were performed by using the SPSS 22.0 software (SPSS Inc., Chicago, IL, USA).

### 2.7. Demographic Data

From the five hundred fifty-eight patients that underwent major anatomical lung resection for NSCLC, in the above-mentioned period, forty-eight patients were excluded, leaving a total of five hundred ten patients stratified into two groups.

In the elderly group (median age: 74 years, range: 70–85), 135 patients were included. In this group, 122 patients were males (90.4%) and 13 females (9.6%).

In the control group (median age: 62 years, range: 50–70), 375 patients were included. In this group, 339 patients (90.4%) were males and 36 (9.6%) were females.

### 2.8. Preoperative Results

Regarding the preoperative characteristics of the two groups, the following results were noted. As expected, elderly patients had cumulative more preoperative comorbidities (74.1% vs. 53.3%, *p* = 0.001) than the control group. Arterial hypertension (42.2% vs. 26.9%, *p* = 0.001) and atrial fibrillation (10.4% vs. 3.7%, *p* = 0.005) were more frequent among elderly patients. However, comorbidities such as COPD, diabetes mellitus, ischemic heart disease/coronary artery disease (IHD-CAD), acute myocardial infarction (AMI), cerebral vascular disease, history of tuberculosis, other malignancy, systemic collagen disease, and preoperative chemotherapy-radiotherapy were similar in both groups. The demographic and preoperative clinical data are summarized in Table 1.

### 2.9. Mortality and Follow-Up

An outpatient follow-up was performed up to the 30th postoperative day. For this reason, mortality and morbidity were calculated up to this date.

## 3. Results

### 3.1. Surgical Data

No statistical significance was detected concerning the extent of the lung resection (*p =* 0.121) among the two groups. In the control group, 11 segmentectomies, 198 lobectomies, 25 bilobectomies and 141 pneumonectomies were performed. In the elderly group, 5 segmentectomies, 86 lobectomies, 8 bilobectomies, and 36 pneumonectomies were performed. Lobectomy was the most frequent resection chosen in both groups. The data are summarized in Table 2.

### 3.2. Tumor Histology and Differentiation

Squamous cell carcinoma (SCC) was more frequently diagnosed in elderly patients (59.3% vs. 51.5%,), while adenocarcinoma was more often in younger ones (37.1% vs. 25.2%) (*p* = 0.037). Large cell carcinoma or other lung cancers were comparable in both groups.

Regarding tumor differentiation, older patients exhibited higher differentiated tumors at the time of surgery than their younger counterparts (12.6% vs. 6.4%). The same pattern was observed in favor of the elderly group concerning middle tumor differentiation (49.6% vs. 42.7%) (*p* = 0.014). On the other hand, younger patients were diagnosed with lower tumor differentiation at the time of resection (43.5% vs. 29.6%). Undifferentiated tumor rates were comparable for both groups (see Table 2).

### 3.3. Tumor Staging

At the time of operation, the elderly group exhibited tumors of an earlier stage compared with patients of the control group (for stage I: 55.6% for elderly patients vs. 36.6% for the control group, *p* = 0.002), which were more likely to be diagnosed with an increased tumor stage (for stage III: 19.3% for elderly vs. 29.1% for control) (see Table 2). In both groups, the rates of patients diagnosed in stages II and IV were comparable.

### 3.4. Mediastinal Lymph Node Staging

Mediastinal lymph node metastases (N2+) were more often diagnosed in younger patients (N2+: 21.6% vs. 9.6%, *p =* 0.002), while elderly patients exhibited no lymph node metastases (N0) or showed positive N1 disease (N1+). The data are summarized in Table 2.

### 3.5. Postoperative Morbidity and Hospital Stay

Concerning the postoperative complications, the elderly group showed to be more vulnerable to postoperative pneumonia (3.7% vs. 0.8%, *p* = 0.034) and to lung atelectasis (7.4% vs. 2.9%, *p* = 0.040) than the control group.

On the other hand, the diagnosis of postoperative empyema showed statistical significance in favor of the control group (3.2% vs. 0%, *p* = 0.042).

Moreover, the rate of postoperative atrial fibrillation was higher (8.9% vs. 5.6%, *p* = 0.220) among elderly patients, although no statistical significance was shown.

Postoperative complications such as acute myocardial infarction, admission to the ICU and/or re-intubation, ARDS appearance, acute renal failure, incidence of bronchopleural fistula, TIA-CVA, and prolonged air leak from the chest tube were comparable. The data are summarized in Table 3.

Finally, regarding the number of reoperations performed, the need for a second surgical revision for postoperative bleeding (*p* = 0.601) was similar among the groups. What was found to be of significance was the need for reoperation for another reason, (other than hemorrhages, e.g., prolonged air leak) in favor of the control group (5.3% vs. 0%, *p* = 0.003).

Elderly patients showed a trend towards increased postoperative morbidity after bilobectomy (50% vs. 20% for younger patients, *p* = 0.6). However, no statistical significance was found. All the anatomical lung resections, even for the pneumonectomy morbidity rates, were comparable (see Table 4).

The severity of the complications recorded in the elderly group was not affected by the extent of lung resection. Concerning major complications, no statistical significance was detected between pneumonectomy and lobectomy among elderly patients (major complications: 2.8% for pneumonectomy vs. 5.8% for lobectomy, *p* = 0.651). Similar were the findings for minor complications. The data are summarized in Table 5.

Hospital stay was similar in both age groups (7.9 ± 5.8 days in the control group vs. 7.8 ± 5.4 in the elderly, *p* = 0.8).

### 3.6. Postoperative Mortality

Elderly patients showed a slightly increased rate of 30-day mortality in comparison with younger ones (5.2% for the elderly vs. 2.7%). However, no statistical significance was detected (*p* = 0.168). In addition, among the elderly population, postoperative mortality was not associated with the extent of the operation. Pneumonectomy in elderly patients was not associated with increased mortality in comparison to operations with lesser parenchymal resection (30-day mortality 2.9% for pneumonectomy vs. 4.7% for lobectomy, *p* = 0.07) (See Table 5).

### 3.7. Univariate Analysis

In order to identify the factors that have independent significance on overall survival, a univariate Cox regression analysis was carried out for both groups. As possible risk factors, the preoperative comorbidities of the patients were evaluated. The following preoperative comorbidities were evaluated: hypertension, COPD, diabetes, IHD-CAD, AMIN, atrial fibrillation, brain vascular disease, TBC, other malignancy, collagen disease, and preoperative radiochemotherapy. For the control group, the addition of preoperative chemo or radiotherapy to downstage the patients and COPD were identified as independent factors for a worse prognosis. In the elderly group, the only factor that was found to be of importance was the existence of COPD (Table 6).

### 3.8. Survival

**Figure 1:**
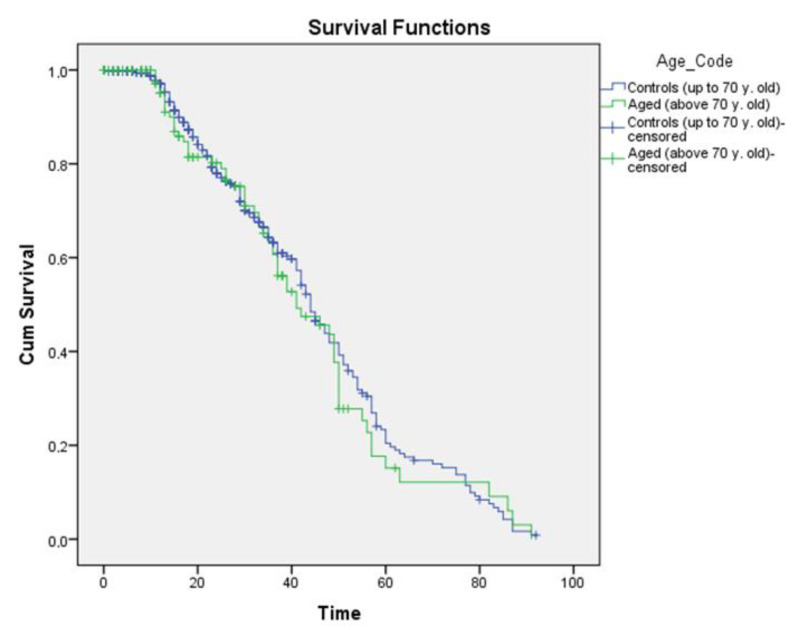
Overall survival.

Elderly patients showed a mean overall survival of 43.4 months after lung resection. In this age group, 3- and 5-year survival were 46% and 20.4%, respectively. In the control group, the mean survival was 45.3 months. Here, the 3- and 5-year survival were 48% and 25%, respectively. No statistical significance concerning survival was detected between the two groups (*p* = 0.579) (Figure 1).

**Figure 2:**
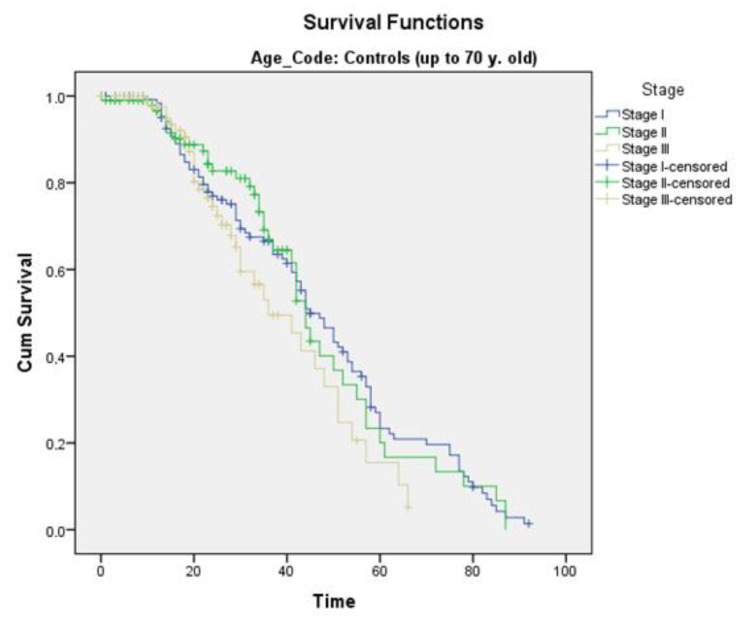
Survival according to the stage in the control group. Mean survival for stage I: 46.8 months, stage II: 46.4 months, and stage III: 39.2 months. (Blue: stage I, green: stage II, gold: stage III).

**Figure 3:**
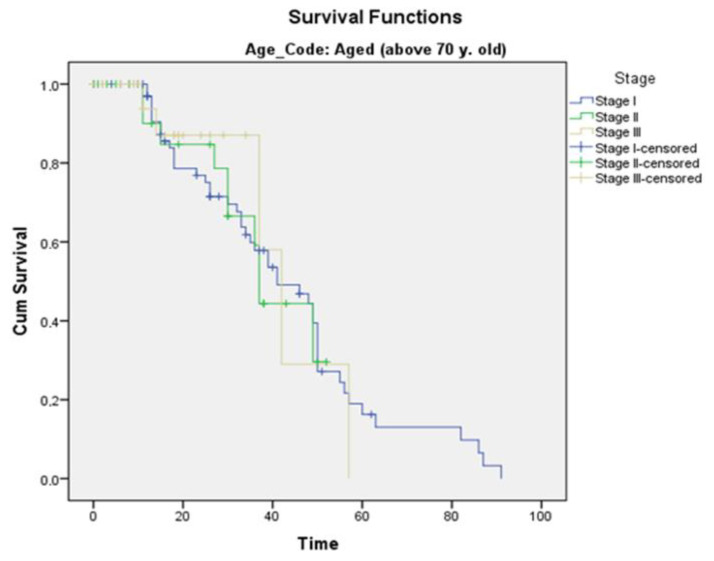
Survival according to the stage in the elderly group. Mean survival for stage I: 43.3 months, stage II: 41.0 months, and stage III: 39.9 months. (Blue: stage I, green: stage II, gold: stage III).

Furthermore, survival was comparable for both age groups regarding the tumor stage. For stage I patients, the elderly group showed a mean survival of 43.3 months compared to 46.8 months in the control group. For stage II patients, the elderly group exhibited a mean survival of 41.0 months in comparison to 46.4 months in the control group. Finally, stage III patients showed a mean survival of 39.9 months among the elderly in comparison to 39.2 months among the younger ones (Figure 2 and Figure 3).

N2 disease in both groups showed reduced survival. Elderly patients with positive N2 disease showed a mean survival of 37.3 months in comparison with 43.5 months for elderly patients with N0 or N1+ disease (*p* = 0.953). Furthermore, in the control group, N2 disease showed a reduced survival of mean 37.8 months in comparison with 46.3 months for an N0 or N1+ disease (*p* = 0.056). However, in both groups, no statistical significance was detected.

## 4. Discussion

Surgery is the treatment of choice in early-stage non-small cell lung cancer (NSCLC), whatever the age, since it offers the best potential for a cure, as is the case with most solid tumors [15]. In the 1970s, the prevailing opinion was that thoracotomy at the age beyond 70 years was prohibitive since initial reports presented high postoperative mortality rates, close to 20%, and considered surgery too dangerous [16]. Over the next two decades, as life expectancy rise, surgeons came across growing numbers of elderly patients who were presented with anatomically and ontologically correctable pathology. However, lung surgery in elderly patients could be challenging [4,13]. Finishing our study, we came up with interesting results and facts that can answer the question if open lung resection in elderly NSCLC patients is justified.

Our first finding was the fact that older patients presented with increased comorbidities than the controls related to increasing age (74.1% vs. 53.3%, *p* = 0.001). Hypertension and cardiac comorbidities such as atrial fibrillation were in favor of the elderly group.

Furthermore, two significant verifications were made concerning tumor behavior and characteristics. Geriatric patients are associated with better long-term prognoses because of a lower incidence of adenocarcinoma and a higher incidence of squamous cell tumors. Dexter et al. reported an incidence of 28–71% for the squamous subtype in elderly patients undergoing surgery [17]. Massard et al. and DeMaria et al. reported even higher rates of 70% and 71%, respectively [18,19]. In addition, in the above studies, the rates of adenocarcinoma in elderly patients were lower (7–34%) [17,18,19]. These findings were in concordance with our results. We also observed an increased percentage of the squamous cell subgroup among the elderly (59.3%), whereas adenocarcinoma was more frequent among younger patients (37.1%). No trend was found for large-cell undifferentiated carcinoma and carcinomas not otherwise specified, as also previously published [20].

Another observation was that elderly patients exhibited better tumor differentiation (well and middle-differentiated tumors) at the time of surgery than the control, whereas younger patients exhibited poorly differentiated ones. No age preference was observed for undifferentiated tumors. In the current study, statistical significance concerning tumor staging of NSCLC was observed. Elderly NSCLC patients were diagnosed at an earlier stage (stage I), in contrast to younger patients who presented at a more advanced stage. Furthermore, younger patients were more likely to be diagnosed with mediastinal lymph node metastases (positive N2+ disease) than elderly patients. The explanation is twofold. Older patients have the tendency to visit their practitioner more often than younger ones. Additionally, lung cancer among geriatric patients shows a clinically reduced growth rate and metastatic potential, with an apparently inverse relationship between stage and age [19,20,21,22].

Elderly patients that are undergoing open lung resection for lung cancer show an increased postoperative morbidity and mortality than younger ones [23]. Romano et al. showed an association between increased mortality and advanced age. In a large series of 12,439 patients undergoing lung cancer resection, they demonstrated that the strongest risk factor for death was advanced age above 79 years, having adjusted the odds ratio almost three times that of non-elderly [24]. Over the last decades, many studies have also demonstrated such an effect of increased mortality associated with senescence [25,26]. On the other hand, others found no causal relationship [27,28], although Harvey et al. reported that operative mortality did not increase significantly until the age of 80 years [29]. Whittle et al. concluded that postoperative mortality increases with increasing age [30]. In our study, although the elderly group presented with an increased number of preoperative comorbidities, as already mentioned, this was not associated with the increased rate of postoperative complications, increased hospital stay, or an increase in 30-day mortality.

The extent of surgery is another important factor that influences the overall rates of morbidity and mortality rates in the elderly. Although lobectomy comprises the most frequent procedure performed [31], however, pneumonectomy may be needed in some cases of lung cancer in the elderly [32], indicating a more extensive disease that required more extensive surgery. We observed a low mortality rate (5.2%) among the elderly group, even with extended resections. Moreover, we found that the total morbidity rates among the elderly were relatively low (28.1% vs. 26.1% for control), even when extended resections were included. Postoperative complications, regardless of the extent of the lung resection, were comparable in both groups. We believe that these findings lay on the careful selection of patients and on the fact that preoperative comorbidities were treated with the appropriate medication before the operation.

Pneumonectomy carries a higher risk of mortality, according to previously published data [22,33], although some have found no difference between pneumonectomy and lobectomy [34]. The use of pneumonectomy should not be ruled out in the elderly, but call for a careful and balanced approach, starting with a meticulous preoperative pulmonary and cardiovascular assessment. Especially concerning pneumonectomy in the elderly, we believe that our low mortality rate is attributed to the very careful selection of patients, as evidenced by the lower frequency of pneumonectomy in this age group (26.7% in the elderly group vs. 37.6% in the control group). Lung-sparing procedures enable the preservation of pulmonary function and could decrease operative morbidity and mortality in patients with lung cancer [35]. We believe that lung-sparing resection for an early lung cancer stadium should be preferred. In this case, however, a possible higher recurrence rate should be taken into account [36,37].

Regarding postoperative events in the current study, postoperative pneumonia or lung atelectasis is more possible to occur in elderly patients than in younger ones (3.7% vs. 0.8% and 7.4% vs. 2.9%), respectively [38]. Shiono et al., Simonsen et al., and Wang et al. also identified old age as a risk factor for empyema, postoperative pneumonia, and lung atelectasis [38,39,40]. We believe that elderly patients, after a lung operation, should be mobilized early and should undergo intensive pulmonary physiotherapy perioperatively. Combined perioperative inhalation therapy and physiotherapy in elderly patients can improve lung function and reduce the incidence of perioperative complications [41]. In addition, an improved result after lung resection in elderly patients, especially in those with COPD and cigarette smoking, could also be achieved through pulmonary training and physiotherapy [42].

We identified COPD in elderly patients, after lung resection for NSCLC, as an adverse prognostic factor for survival. Sekine et al. showed similar results in patients with COPD undergoing lung resection. In this study, age and COPD were identified as prognostic factors for survival. In addition, COPD was identified as an adverse factor for survival and pulmonary complication. Generally, 50–80% of patients diagnosed with lung cancer have pre-existing COPD. The coexistence of COPD can worsen survival because of the possible inadequate treatment of lung cancer, poorer pulmonary function, and reduced quality of life [43]. We believe that the coexistence of COPD, with other comorbidities in elderly patients, led to reduced patients’ general condition and survival. Similar findings were reported in the meta-analysis of Xu et al. In their study, among 14.171 patients operated for NSCLC, COPD was identified as a risk factor for poorer survival, a higher risk for bronchopleural fistula, pneumonia, prolonged air leak, and prolonged mechanical ventilation [44].

In the literature, another limitation of surgery, as a therapeutic approach, constitutes those geriatric patients presenting with tumors extending beyond stage I. Long-term survival in cancer patients with nodal (N2) or T3N0 disease showed disappointing results. Similarly, Riquet et al. reported a median survival of only 11 months in N2 patients over 75 years of age, in contrast to 20% 5-year and 18-month median survivals in those younger than 75 [45].

Lung resection in selected elderly patients could prolong survival. The 5-year overall survival rate after lung resection in the elderly population ranges between 25 and 58%. In our study, the 3-year overall survival rate was 46% and 48% among the elderly and control group respectively. Elderly patients showed a slightly reduced 5-year survival rate (20.4% vs. 25%). However, no statistical significance was shown. We believe that this difference in survival between the two populations lies in the natural course of elderly patients. Morandi et al., in a study of 85 patients aged between 70 and 88 years old undergoing lung resection, reported a 5-year overall survival rate of 28% [31]. Massad et al. reported a 5-year overall survival rate of 33% for 223 patients aged between 70 and 84 years [18]. Similar were the results of Okami et al. for older patients (between 80 and 92 years old) [46]. Our opinion stands for the careful selection of elderly patients who can be treated surgically in order to decrease postoperative morbidity and mortality [47,48]. Concerning the low 5-year survival rate for stage I in both age groups (20% vs. 25%), we believe that it should be attributed to a possible preoperative selection bias. We believe that in our study, which investigated lung resections with an open thoracotomy, we included more patients with locally advanced lung cancer, more complicated cases, and more comorbid patients. For this reason, we believe that survival was equally reduced in both groups. On the other hand, we believe that cases with less complex cases or with tumors on the periphery of the lung were removed with video-assisted thoracic surgery.

### Limitations

Our study is firstly limited by its retrospective nature. In addition, in the study limitations, a possible selection bias should be included. Elderly patients with reduced general condition or lymph node metastases would not be referred for surgery. Furthermore, the limitations of the study should be included in the definition of COPD only with functional criteria. Other factors, such as radiological findings and clinical criteria, were not considered. In addition, due to the retrospective nature of our study, the patient’s habits, such as alcohol consumption or educational level, could not be evaluated. As a result, further comparison of the comorbidity status of each group, for example with the Charlson Risk Index score, could not be possible. In addition, due to the above restriction, the evaluation of intraoperative findings, such as the localization of the lung tumor (central tumor vs. tumor in the periphery), was not possible. Moreover, the numerical difference between the groups could have influenced the results of the study. Furthermore, an additional limitation of our study was the short postoperative follow-up of the patients. The outpatient follow-up was limited to the 30th postoperative day. As a result, a longer observation of the results after major lung resections, such as pneumonectomy, was not possible. We believe that the findings of the current study should be interpreted cautiously as single-center observations and no definitive conclusions should be drawn. However, our study of 510 patients, including 135 older than 70 years undergoing an open surgery, represents a reflection of the clinical reality. The authors of the current study believe that elderly patients are ideal candidates for minimally invasive lung resection. However, in selected elderly patients, open major lung resection through thoracotomy could be performed safely with good postoperative and oncological results.

## 5. Conclusions

To answer the paper’s main question, we can synopsize the following. Elderly patients with NSCLC present with increased comorbidities than their younger counterparts. The squamous cell carcinoma subtype is more frequently observed, which carries a better prognosis than lung adenocarcinoma seen in younger patients. Additionally, older patients have the tendency to present with better differentiated tumors, and in an earlier stage than younger ones. Because of the nature of lung cancer among geriatric patients, they usually exhibit a clinically reduced growth rate and metastatic potential. Therefore, major lung resections in selected elderly patients may be associated with an increased probability to prolong survival. The fact that postoperative morbidity and mortality also increased in concordance with senescence was not observed in our study and is considered a myth. The same stands for postoperative complications and overall survival. Surprisingly, we observed no differences between both groups regarding hospital stay and major or minor postoperative complications, even with extended operations such as pneumonectomies. We also concluded that the extent of surgery among the elderly group was not a prognostic factor for adverse outcomes. The only parameter that negatively influences the outcome was COPD. These patients should be mobilized early and intensive pulmonary physiotherapy should take place perioperatively in order to avoid postoperative atelectasis and pneumonia. Therefore, major lung resection in selected elderly patients with NSCLC is justified and radical surgery, even with open thoracotomy, should be offered as a therapeutic option in order to prolong life expectancy.

## Figures and Tables

**Table 1:** Preoperative characteristics of the study groups.

Variable	Control Group	Elderly Group	*p*-Value
	*n* (%)	*n* (%)	
**Total (*n*)**	*n* = 375	*n* = 135	
Sex male/female	339/36	122/13	
Hypertension	101 (26.9%)	57 (42.2%)	0.001
COPD	67 (17.9%)	33 (24.4%)	0.102
Diabetes mellitus	43 (11.5%)	15 (11.1%)	0.526
IHD-CAD	36 (9.6%)	21 (15.6%)	0.45
AMI	5 (1.3%)	1 (0.7%)	0.499
Atrial fibrillation	14 (3.7%)	14 (10.4%)	0.005
Brain vascular disease	7 (1.9%)	6 (4.4%)	0.999
TBC	7 (1.9%)	4 (3.0%)	0.327
Malignancy other	26 (6.9%)	8 (5.9%)	0.431
Systemic autoimmune diseases	6 (1.6%)	3 (2.2%)	0.44
Preop. chemo-radiotherapy	26 (6.9%)	5 (3.7%)	0.212
Total number of comorbidities	53.30%	74.10%	0.001

Abbreviations: COPD: chronic obstructive pulmonary disease, IHD-CAD: ischemic heart disease -coronary artery disease, AMI: acute myocardial infarction, TBC: tuberculosis.

**Table 2:** Surgical characteristics, histological type, and survival postoperative.

Variable	Elderly (*n* Patients, %)	Control (*n* Patients, %)	*p* Value
Resektion Type			*p* = 0.121
Pneumonectomy	36 (26.7%)	141 (37.6%)	
Bilobectomy	8 (5.9%)	25 (6.7%)	
Lobectomy	86 (63.7%)	198 (52.8%)	
Segmentectomy	5 (3.7%)	11 (2.9%)	
Tumor differentiation			*p =* 0.014
High	17 (12.6%)	24 (6.4%)	
Middle	67 (49.6%)	160 (42.7%)	
Low	40 (29.6%)	163 (43.5%)	
Undifferent	11 (8.1%)	28 (7.5%)	
Tumor histology			*p =* 0.037
SCC	80 (59.3%)	193 (51.5%)	
Adenocarcinoma	34 (25.2%)	139 (37.1%)	
Large cell carcinoma	14 (10.4%)	35 (9.3%)	
other	7 (5.2%)	8 (2.1%)	
Stage			*p =* 0.002
I	75 (55.6%)	136 (36.6%)	
II	30 (22.2%)	112 (29.9%)	
III	26 (19.3%)	109 (29.1%)	
IV	4 (3.0%)	18 (4.8%)	
N-Status			*p =* 0.002
N0–1	122 (90.4%)	294 (78.4%)	
N2	13 (9.6%)	81 (21.6%)	
30-day mortality	7 (5.2%)	10 (2.7%)	*p =* 0.168
Survival			*p =* 0.57
3-y-s	46%	48%	
5-y-s	20.4%	25%	
Mean (months)	43.469	45.298	
Survival pro stage (mean survival in months)			
Stage I	43.317	43.317	*p =* 0.5
Stage II	46.442	41.089	*p =* 0.6
Stage III	39.209	39.912	*p =* 0.6

**Table 3:** Postoperative complications.

Complication	Control	Elderly	*p*-Value
	(*n* Patients, %)	(*n* Patients, %)	
**AMI**	1 (0.3%)	1 (0.7%)	0.46
ICU-admission/Re-intubation	13 (3.5%)	5 (3.7%)	1
ARDS	5 (1.3%)	2 (1.5%)	1
Re-operation for Hemorrhage	13 (3.5%)	6 (4.4%)	0.601
Re-operation (other reasons)	20 (5.3%)	0 (0%)	0.003
Acute renal failure	2 (0.5%)	3 (2.2%)	0.119
Empyema	12 (3.2%)	0 (0%)	0.042
Bronchopleural fistula	3 (0.8%)	0 (0%)	0.569
TIA-CVA	3 (0.8%)	0 (0%)	0.569
MOF	2 (0.5%)	1 (0.7%)	1
Pneumonia	3 (0.8%)	5 (3.7%)	0.034
Atelectasis	11 (2.9%)	10 (7.4%)	0.04
Atrial Fibrillation	21 (5.6%)	12 (8.9%)	0.22
Prolonged air leak	29 (7.7%)	12 (8.9%)	0.712

AMI: acute myocardial infarction, ICU: intensive care unit, ARDS: Acute Respiratory Distress Syndrome, TIA-CVA: transient ischemic attack-cerebrovascular accident, MOF: multiple organ failure.

**Table 4:** Complications according to the extent of the lung resection.

Type of Operation/ Complications	Elderly (*n*, %)	Control (*n*, %)	*p* Value
Pneumonectomy	9 (25%)	37 (26.2%)	0.5
Bilobectomy	4 (50%)	5 (20%)	0.6
Lobectomy	24 (27,9%)	52 (26.3%)	0.6
Segmentectomy	1 (20%)	5 (45.5%)	0.2
Total number of complications	38 (28.1%)	99 (26.4%)	0.2

**Table 5:** 30-day mortality and morbidity among the elderly group in comparison with the extent of parenchymal resection.

Variable	Pneumonectomy (*n*, %)	Lobectomy (*n*, %)	*p*-Value
30-day mortality/No. Cases (%)	1 (2.9%)	4 (4.7%)	0.07
Major complications	1 (2.8%)	5 (5.8%)	0.651
Minor complications	7/36 (19.4%)	19/86 (22.1%)	0.160

**Table 6:** Univariate analysis among patient population.

	Parameter	Hazard Ratio (95% CI)
Age < 70 years old	Preoperative chemo-/ radio-therapy	0.350 (0.172–0.715)
	COPD	1.661 (1.131–2.438)
Age > 70 years old	COPD	1.756 (1.004–3.073)

## Data Availability

The data presented in this study are available upon request from the corresponding author.
